# Cost-effectiveness and cost-utility analyses of hospital-based home care compared to hospital-based care for children diagnosed with type 1 diabetes; a randomised controlled trial; results after two years’ follow-up

**DOI:** 10.1186/s12887-016-0632-8

**Published:** 2016-07-15

**Authors:** Irén Tiberg, Björn Lindgren, Annelie Carlsson, Inger Hallström

**Affiliations:** Department of Health Sciences, Lund University, SE-221 00 Lund, Sweden; Department of Health Sciences, Lund Universit, Lund, Sweden; National Bureau of Economic Research (NBER), Cambridge, MA USA; Department of Paediatrics, Skåne University Hospital, Lund, Sweden; Department of Health Sciences, Lund University, Lund, Sweden

**Keywords:** Metabolic control, Quality of life, Direct and indirect costs, Cost-utility, Cost-effectiveness

## Abstract

**Background:**

Practices regarding hospitalisation of children at diagnosis of type 1 diabetes vary both within countries and internationally, and high-quality evidence of best practice is scarce. The objective of this study was to close some of the gaps in evidence by comparing two alternative regimens for children diagnosed with type 1 diabetes: hospital-based care and hospital-based home care (HBHC), referring to specialist care in a home-based setting.

**Methods:**

A randomised controlled trial, including 60 children aged 3–15 years, took place at a university hospital in Sweden. When the children were medically stable, they were randomised to either the traditional, hospital-based care or to HBHC.

**Results:**

Two years after diagnosis there were no differences in HbA1c (*p* = 0.777), in episodes of severe hypoglycaemia (*p* = 0.167), or in insulin U/kg/24 h (*p* = 0.269). Over 24 months, there were no statistically significant differences between groups in how parents’ reported the impact of paediatric chronic health condition on family (*p* = 0.138) or in parents’ self-reported health-related quality of life (*p* = 0.067). However, there was a statistically significant difference regarding healthcare satisfaction, favouring HBHC (*p* = 0.002). In total, healthcare costs (direct costs) were significantly lower in the HBHC group but no statistically significant difference between the two groups in estimated lost production (indirect costs) for the family as a whole. Whereas mothers had a significantly lower value of lost production, when their children were treated within the HBHC regime, fathers had a higher, but not a significantly higher value. The results indicate that HBHC might be a cost-effective strategy in a healthcare sector perspective. When using the wider societal perspective, no difference in cost effectiveness or cost utility was found.

**Conclusions:**

Overall, there are only a few, well-designed and controlled studies that compare hospital care to different models of home care. The results of this study provide empirical support for the safety and feasibility of HBHC when a child is diagnosed with type 1 diabetes. Our results further indicate that the model of care may have an impact on families’ daily living, not only during the initial period of care but for a longer period of time.

**Trial registration:**

ClinicalTrials.gov with identity number NCT00804232, December 2008.

## Background

During the past decade, a rapidly increasing incidence of type 1 diabetes has been reported from many parts of the world with a shift towards a younger age of onset [1, 2]. In Sweden, the incidence rate has risen from 21.6 per 100.000 in 1978–1980 to 43.9 in 2005–2007; recent data suggests a break in the increasing trend, but these findings need to be confirmed over a longer period of time [3]. It is a serious disease for the child, and it has significant implications also for the family as regards daily activities, work and social life [4–6].

The disease also has substantial economic consequences over time, both for individuals and society [7–11]. People, diagnosed with diabetes during childhood, have been seen to be disadvantaged in adult employment, with lower earnings and lesser probability of attaining the highest level of education, although late complications appear to be the most important determinant of social consequences in later life [8–11]. The findings emphasize the importance of choosing an appropriate strategy for handling diabetes already at the time of diagnosis.

The diagnosis of childhood diabetes represents a major stressor event for parents [12–14] and parents approach a challenging process of changes in the patterns of daily activities [15]. Conventionally, a child newly diagnosed with type 1 diabetes has been admitted to hospital as part of his or her initial management, partly for child-safety reasons, partly based on the belief that the family needs time to adjust to the requirements of the disease [16, 17]. Internationally, there is a considerable variation in length of hospitalization at diagnosis but with a trend towards shorter lengths of stay or exclusively outpatient management [18–22]. In the United Kingdom, for instance, children with newly diagnosed type 1 diabetes are increasingly treated exclusively at home from diagnosis and onwards [5]. To obtain safe care in a home-management environment requires adequate expert support for parents, not least during the first days after diagnosis, when parents still have limited knowledge and understanding of the situation and its potential risks [5]. Existing evidence is insufficient regarding the consequences of alternative models of initial management, both in perspectives of child, parents, health services, and society [5, 12, 23]. Information on outcomes in relation to resource use is necessary when strategic decisions on the allocation of scarce healthcare resources are made [24]; it is required by government bodies in a large number of countries [25–27].

The objective of this study was to close some of the gaps in evidence by comparing two alternative regimens for children diagnosed with type 1 diabetes: hospital-based care and hospital-based home care (HBHC), referring to specialist care in a home-based setting. We have previously reported on metabolic control, health-care satisfaction and health-care costs one month after diagnosis [28]. In this paper, the focus is on metabolic control, health-related quality of life, direct (i.e., healthcare) costs, and indirect costs (i.e., productivity losses) two years after diagnosis. Costs are related to metabolic control of the child’s disease (cost-effectiveness analysis) and to health-related quality of life of the parents (cost-utility analysis).

## Methods

The study design was based on the British Medical Research Council framework for development and evaluations of Randomised Controlled Trials (RCT) for complex interventions [29, 30], and has been described in detail elsewhere [31]. The study follows the Consolidated Standards of Reporting Trials (CONSORT) recommendations [32]. Statistical power calculation included the primary outcome HbA1c two years from diagnosis. In order to show a mean difference of 10.5 mmol/mol (Mono S: one percent) between two groups with the power of 0.80 at a significance level of 5%, it would take 30 children in each group. Randomisation in two strata – (a) younger than eight years and (b) eight years and above – was performed by an independent centre for clinical research, using the software R-2.6.1 [33]. The investigators received two sets of coded, sealed, opaque envelopes, one for younger children and one for older children.

### Setting

**Fig. 1:**
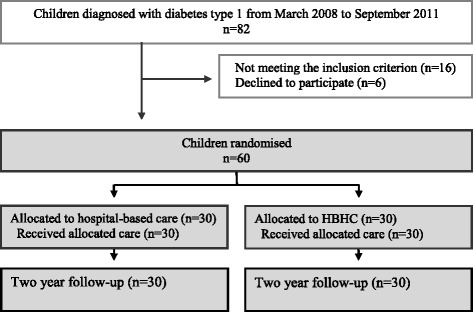
Flow chart of the progress through the phases of the trial two years after diagnosis

The study took place from 1 March 2008 up to the end of August 2011 at the Skåne University Hospital, division of paediatrics at the Children’s Hospital in Lund, Sweden. The study included children, aged 3–15 years and newly diagnosed with type 1 diabetes. The age range of 3–15 was chosen to be as representative as possible of the forms of care normally in practice. However, children younger than three years were not included for the sake of their safety. The follow-up of two years set the upper age limit to 15 as the transition to the adult diabetes care setting when the adolescents have had their 18th birthday. Additional inclusion criteria were that the child did not have any other difficult chronic illness, had no sibling with type 1 diabetes, was not in social-care custody, and lived in a family who could understand and speak the Swedish language. When the child was medically stable, he or she received subsequent care according to the randomisation procedure; either continued hospital-based care or HBHC. After the first month, all families followed the conventional care with visits at the outpatient department unit. A flow chart through the phases of the trial up to the two-year follow-up in September 2013 is shown in Fig. 1.

### Hospital-based care

Children randomised to hospital-based care followed the conventional care according to the Swedish national guidelines for paediatric diabetes [16], which involved 1–2 weeks [28] of hospital-based care, while child and parent had educational sessions with the diabetes team members. The information followed a check-list, where each discipline was responsible for different portions of information. One parent could stay at the hospital with the child during the night and the other parent was encouraged to be present during the educational sessions. When the family had received most of the planned information, they were able to be on a short leave from the hospital before the child was actually discharged. For children of school age, the diabetes nurse offered to make a school-visit with the purpose of informing teachers and school-friends about diabetes and insulin treatment in addition to the outpatient visits offered by the physician. Families in the hospital-based group had access to telephone support from the diabetes nurse during working-hours five days a week. During evenings, nights and week-ends, they could receive assistance from the general hospital staff.

### Hospital-based home care

Children randomised to HBHC left the Children’s Hospital together with their parents, when the child was medically stable, and stayed at a Family House, placed in the hospital area, up to a week [28]. The family house, supported by a non-profit Child foundation, offers sick children and their families a home-like environment when the child is under care at the hospital. The stay included support of a diabetes nurse during parts of the day. Information meetings with other professionals in the diabetes team were held at the Children’s Hospital in accordance with the conventional care. The contents of the information given to families were the same in both groups. The active parts of the HBHC were defined as an individualised learning process through supportive interaction between the family and the diabetes nurse at the Family House. Another active part included the home-like environment which allowed families to practise the diabetes management with the concurrent support. The final active part was an increased support after discharge in the form of three home and/or school visits by the diabetes nurse besides the regular diabetes check visits as well as increased telephone access to the diabetes nurse during day and evening, seven days a week. During nights they could receive assistance from the general hospital staff.

### Health outcome measurements

Outcomes included extensive data from valid and reliable instruments and depending on type of outcomes, data were collected at the time of discharge and at six, 12 and 24 months from diagnosis. Assessments included metabolic control measured by glycated haemoglobin (HbA1c), episodes of severe hypoglycaemia (defined as unconsciousness with or without cramp), insulin regime (multiple subcutaneous injections/insulin pump therapy) and insulin U/kg/24 h. Assessments also included The PedsQL™ Family Impact Module [34, 35] measuring parents’ reported impact of paediatric chronic health condition on the family, and The PedsQL™ Healthcare Satisfaction Generic Module [36, 37], evaluating parents’ satisfaction with healthcare. Both PedsQL™ instruments are scored on a 5-point (0–4) Likert-type scale for the response categories. Parents reported health-related quality of life was assessed by the Swedish SF-36 [38–41]. The instrument includes 36 items measuring parents reported physical and mental health and a single item of parents reported health transition, compared to one year ago. A research assistant, who was not involved in care, collected the outcomes; and parents were instructed to fill in the forms independently of each other.

HbA1c values and insulin units/kg/24 h were registered every third month. For cases without recorded values at the designated time point of six, 12 and 24 months after diagnosis, values were weighted and interpolated between the two closest registrations. Episodes of severe hypoglycaemia and the number of children who changed insulin regime from multiple injections to pump therapy were summed for the two years follow-up. Insulin U/kg/24 h is presented from one time point (24 months from diagnosis). The PedsQL™ scales were linearly transformed into 0 to 100 scales as to facilitate interpretation of the scores [36] and scale scores were computed as the sum of the items divided by the number of item answered [37]. For the PedsQL™ Family Impact Module, higher scores indicate better functioning and thus less negative impact on the family and for the PedsQL™ Healthcare Satisfaction Generic Module, higher scores indicate greater satisfaction.

For the SF-36, each question raw scores were coded, recalibrated in two instances, summed, and transformed into a scale from 0 (worst possible health state measured) to 100 (best possible health state) following the standard SF-36 scoring algorithm [41]. Responses to the SF-36 were used in order to produce utilities for the cost-utility analysis, employing an accepted and validated algorithm for SF-6D [42, 43]. Thus, the number of dimensions was reduced from eight to six, including totally 11 items; responses on items were weighted according to the algorithm, producing scores between minus one and plus one [42, 43]. Plus one is usually interpreted as “perfect health”; zero as the equivalent of “being dead”; negative values as states worse than being dead.

### Resource use measurements

Resource use measurements included both direct and indirect costs. Direct costs comprised of hospital services and health professional’s time use. Data on resource use was obtained from the hospital patient-administrative system and additional documentation. Cost per unit of resource use was obtained from the administrative price-list employed in between-hospitals transactions in 2011 [44]. Table 1 presents detailed information on type of healthcare resource use and its unit cost. Indirect costs included productivity losses as a result of parents’ and relatives’ absence from work due to illness and treatment of the child. Data on absence and earnings was collected by questionnaires to parents or relatives of the child. Following the human capital approach, the number of days that might have been used in labour-market work was translated into monetary terms by multiplying with the average daily wage rate including employer’s contribution to social insurance and collectively agreed private insurance premium. Individual data on incomes were used for parents; however, due to lack of individual information, an average wage rate was used for relatives and other carers. Mean unit cost per day for fathers, mothers, and relatives are shown in Table 1.

**Table 1:** Unit cost of healthcare resource use and loss of production in SEK 2011 level of prices

Healthcare resource use	
Overnight (per night)	
The Children’s hospital	5758
The Family house	556
Initial education (per hour)	
Paediatrician	936
Diabetes nurse	547
Dietician	468
Social worker	515
Initial telephone support (per 15 min)	
Paediatrician	234
Diabetes nurse	137
Dietician	117
Social worker	129
Outpatient visit (per visit)	
Paediatrician	1074
Other healthcare professionals	430
Home/School visit (per visit)	1290
Group education (per occasion)	430
Emergency visit (per visit)	2010
Loss of production per day	
Parent/Relative (months from diagnosis)	Mean (SD)
Fathers (0–1)	2852 (1373)
Fathers (1–6)	2887 (1222)
Fathers (6–12)	3602 (2950)
Fathers (12–24)	4169 (4178)
Mothers (0–1)	2038 (937)
Mothers (1–6)	2121 (859)
Mothers (6–12)	2189 (1017)
Mothers (12–24)	2328 (947)
^a^Relatives (0–24)	1569

Note: Unit costs were obtained from the Swedish southern regional healthcare price list (year 2011)

Note: USD 1 = SEK 6.50, EUR 1 = SEK 9.03 (year 2011) average exchange rate www.riksbank.se

^a^Based on mean income in Sweden for men and for women

### Statistical methods

Analyses were conducted using SPSSTM (version 22); differences with *p*-values <0.05 were considered statistically significant. Descriptive statistics were used to report on parents’ background characteristics. Continuous variables were checked for distributional characteristics, and since the data were assessed as normally distributed, parametric tests were used [45]. Student’s t-test was used to compare groups in HbA1c, episodes of severe hypoglycaemia, number of children who changed insulin regime from multiple injections to pump therapy and insulin U/kg/24 h two years from diagnosis. Repeated measures ANOVA was used to analyse changes in the child’s metabolic control with HbA1c (at six, 12 and 24 months from diagnosis), in the impact of paediatric chronic health condition on family (at discharge, six, 12 and 24 months from diagnosis), in parents’ healthcare satisfaction (at discharge, six, 12 and 24 months from diagnosis) and in health-related quality of life with SF-6D (at discharge, 12 and 24 months from diagnosis), and over time.

## Results

The children’s medical variables and parent’s background characteristics at diagnosis are presented in Table 2.

**Table 2:** Children’s medical variables and parents background characteristics at diagnosis in hospital based care and HBHC

	Hospital-based care	HBHC
*Children’s medical variables at diagnosis*	*n = 30*	*n = 30*
Boys/Girls, n	16/14	20/10
Age at diagnosis, mean (SD)	8.6 (3.8)	8.8 (3.7)
pH at diagnosis, mean (SD)	7.35 (0.08)	7.35 (0.11)
HbA1c (mmol/mol), mean (SD)	87.3 (28.7)	85.7 (26.4)
*Parents background characteristics at diagnosis*	*n = 58*	*n = 58*
Mothers age, mean (SD)	40.4 (5.3)	40.1 (6.2)
Fathers age, mean (SD)	43.6 (6.6)	42.6 (5.7)
Education, n (%)		
Mothers with university degree	18 (60.0)	15 (51.7)
Fathers with university degree	13 (46.4)	15 (50.0)
Working hours, n (%)		
Mothers not employed	4 (13.3)	3 (10.3)
Mothers full time	19 (63.3)	12 (41.4)
Mothers part time,	7 (23.3)	14 (48.3)
Fathers not employed	1 (3.6)	0 (0.0)
Fathers full time	27 (96.4)	28 (96.5)
Fathers part time	0 (0.0)	1 (3.5)
Monthly income before tax, mean SEK (SD)		
Mothers	26435 (13606)	22755 (8385)
Fathers	37295 (16550)	32629 (14832)

### Health outcomes

Children’s mean HbA1c; measurements over time and between group comparisons are presented in Table 3. Two years after diagnosis, there were no differences in HbA1c (mmol/mol) with a mean of 53.7 (SD 8.0) in the hospital-based care and 53.1 (8.3) in the HBHC (*p* = 0.777), in episodes of severe hypoglycaemia with a mean of 0.03 episodes in hospital-based care and 0.13 in HBHC (*p* = 0.167), or in insulin U/kg/24 h with a mean of 0.85 (SD 0.30) in the hospital-based care and 0.94 (0.31) in the HBHC (*p* = 0.269). There were no differences in the number of children, who changed insulin regime from multiple injections to pump therapy during the two years’ follow-up with a mean of 0.23 (SD 0.43) in the hospital-based care and 0.27 in the HBHC group (*p* = 0.770).

**Table 3:** Children’s mean HbA1c; measurements over time and between group comparisons

Repeated measures ANOVA	Mean	SD	Significance	95 % Cl
HbA1c between group comparison			*p* = 0.907	
Control group (*n* = 30)	48.5			46.2–51.0
6 month	43.3	5.9		41.0–45.7
12 months	48.6	7.2		45.5–51.7
24 months	53.7	8.1		50.7–56.7
Intervention group (*n* = 30)	48.4			46.0–50.8
6 month	42.8	7.1		40.4–45.2
12 months	49.2	9.7		46.0–52.3
24 months	53.1	8.3		50.1–56.1

Of the total of 116 parents, 63 parents responded to The PedsQL™ Family Impact Module (Table 4), and 58 parents responded to The PedsQL™ Healthcare Satisfaction Generic Module (Table 5) at all the four time points, and 76 parents responded to SF-36 at the three time points, presented as SF-6D in Table 6. In the measurements over time and between group comparisons, there were no statistically significant differences between groups in how parents’ reported the impact of paediatric chronic health condition on family (*p* = 0.138) or in parents’ self-reported health-related quality of life (*p* = 0.067). However, there was a statistically significant difference regarding healthcare satisfaction, favouring HBHC (*p* = 0.002).

**Table 4:** Impact of paediatric chronic health condition on family; measurements over time and between group comparisons

Repeated measures ANOVA	Mean	SD	Significance	95 % Cl
Family impact between group comparison			*p* = 0.138	
Control group (*n* = 29)	71.1	12.5		66.3–75.8
At discharge	65.3	14.3		59.9–70.7
6 month	76.1	13.7		70.9–81.4
12 months	72.5	13.9		67.2–77.8
24 months	76.1	13.7		70.9–81.4
Intervention group (*n* = 34)	75.8	12.3		71.5–80.0
At discharge	67.7	14.1		62.8–72.6
6 month	77.6	14.5		72.5–82.7
12 months	77.4	13.3		62.8–72.6
24 months	80.6	15.7		74.8–85.7

**Table 5:** Parents’ healthcare satisfaction; measurements over time and between group comparisons

Repeated measures ANOVA	Mean	SD	Significance	95 % Cl
Healthcare satisfaction; between group comparison			*p* = 0.002*	
Control group (*n* = 29)	76.2	17.0		69.7–82.7
At discharge	73.1	21.6		64.9–81.3
6 month	77.9	20.9		70.0–85.9
12 months	78.7	17.0		72.3–85.2
24 months	74.9	22.8		66.2–83.6
Intervention group (*n* = 29)	88.4	10.5		84.4–92.5
At discharge	90.7	7.9		87.7–93.7
6 month	90.7	12.1		86.1–95.4
12 months	85.7	15.7		79.7–91.7
24 months	86.7	14.2		81.3–92.0

*p < 0.05

**Table 6:** Parents’ self-reported health-related quality of life (SF-6D), measurements over time and between group comparisons

Repeated measures ANOVA	Mean	SD	Significance	95 % Cl
SF-6D between groups comparison			*p* = 0.067	
Control group (*n* = 40)	0.775			0.749–0.802
At discharge	0.746	0.109		0.710–0.782
12 months	0.774	0.107		0.742–0.807
24 months	0.805	0.102		0.773–0.837
Intervention group (*n* = 36)	0.811			0.783–0.839
At discharge	0.783	0.121		0.745–0.821
12 months	0.832	0.098		0.798–0.866
24 months	0.818	0.100		0.784–0.851

### Resource use

In total, over 24 months, healthcare costs (direct costs) were significantly lower in the HBHC group, Swedish Crown (SEK) 65 464 against SEK 81 676 for the hospital-based care group (Table 7). This is mainly due to the fact that initial care during the child’s first month was significantly lower for HBHC. Home or school visits by a diabetes nurse during the following 23 months were significantly higher for the HBHC group, SEK 5 590 against SEK 2451 for the hospital-based care group. Diabetes-related re-admissions to hospital were also higher for the HBHC group, but statistically insignificant.

**Table 7:** Healthcare costs (direct costs) in hospital-based care and HBHC, respectively, from diagnosis to 24 months after diagnosis

	Hospital-based care (*n* = 30)	HBHC (*n* = 30)	*p*-value*
	SEK	SEK	
	Mean (SD)	Mean (SD)	
The initial care (0–1 month)	64279 (16928)	45022 (7909)	<0.001*
Outpatient visit (1–24 months)			
Paediatrician	9845 (1264)	9379 (1737)	0.240
Diabetes nurse	1820 (1418)	487 (675)	<0.001*
Other professionals (Social worker, Dietician, Psychologist)	1404 (1059)	1161 (620)	0.282
Home/School visit by Diabetes nurse (1–24 months)	2451 (1866)	5590 (5580)	0.005*
Group education by Diabetes nurse and/or Dietician	559 (630)	373 (514)	0.215
Visits in relation to insulin pump introduction	330 (684)	430 (806)	0.605
Diabetes related emergency visits	603 (1075)	335 (927)	0.305
Diabetes related re-admissions	384 (2103)	2687 (12 717)	0.332
Total costs (1–24 months)	17 397 (4210)	20 443 (14 594)	0.277
Total costs (0–24 months)	81 676 (18 779)	65 464 (16 298)	0.001*

Note: USD 1 = SEK 6.50, EUR 1 = SEK 9.03 (year 2011) average exchange rate www.riksbank.se

*p < 0.05

In total, over 24 months, there was no statistically significant difference between the two groups in estimated lost production (indirect costs) for the family as a whole (Table 8). Mothers had a significantly lower value and fathers had a higher, but not a significantly higher value, of lost production, in the HBHC regime than in the hospital regime. When adding direct and indirect costs together, no statistically significant difference was observed (Table 8). The value of lost production depends both on the number of days absent from work and on the unit cost per day. No statistically significant differences between the two treatment groups were detected in the number of days absent from work, neither for the groups in total (both genders), nor for mothers and fathers analysed separately (Table 9). Mothers had significantly more days absent from work than fathers in the hospital group and in the study population as a whole, but no significant difference was detected between fathers and mothers in the HBHC group/Table 9). Fathers had a significantly higher unit cost than mothers both in the HBHC group and in the hospital group (Table 9).

**Table 8:** Loss of production (indirect costs) due to parents’ and relatives’ absence from work related to the child’s diabetes diagnosis and a summary of direct and indirect costs related to the child’s diagnosis

Relation (months from diagnosis)	n	Hospital-based care	n	HBHC	CI of the difference		*p*-value
		SEK Mean (SD)		SEK Mean (SD)	Lower - Upper		
Fathers (0–1)	22	30 606 (45 387)	26	34 490 (36 148)	−27 574	19 806	0.743
Fathers (1–6)	21	12 819 (20 110)	22	34 045 (59 059)	−48 635	6 182	0.123
Fathers (6–12)	23	19 707 (70 008)	20	17 154 (25 000)	−30 810	35 916	0.878
Fathers (12–24)	14	7 956 (10 685)	16	4 527 (9 234)	−4 017	10 877	0.354
Fathers (sum of 0–24)		19 078 (46 102)		24 834 (39 896)	−19 115	7 602	0.396
Mothers (0–1)	26	35 362 (22 138)	28	26 526 (21 121)	−2 978	20 649	0.139
Mothers (1–6)	24	26 482 (52 562)	26	19 050 (31 356)	−16 955	31 819	0.543
Mothers (6–12)	24	22 321 (38 353)	20	5 849 (14 026)	−1 802	34 746	0.076
Mothers (12–24)	16	15 844 (18 559)	16	4 625 (6 926)	857	21 580	0.035*
Mothers (sum of 0–24)		26 046 (36 574)		15 878 (23 358)	1 141	19 196	0.027*
Parents (0–1)	48	33 182 (34 451)	54	30 361 (29 321)	−9 708	15 351	0.656
Parents (1–6)	44	20 563 (41 290)	47	26 474 (46 584)	−24 295	12 472	0.524
Parents (6–12)	47	21 042 (55 509)	40	11 501 (20 811)	−8 937	28 017	0.308
Parents (12–24)	30	12 163 (15 664)	32	4 576 (8 029)	−1 147	14 028	0.022*
Parents (sum of 0–24)	169	22 789 (41 323)	173	20 175 (35 577)	−5 290	10 519	0.516
Relatives (0–24)	5	12 560 (14 346)	13	7 125 (5 031)	−12 182	23 052	0.451
^a^Sum families (0–24)		23 161 (41 316)		20 710 (32 898)	−5 484	10 385	0.544
Sum direct and indirect costs (0–24)	30	220 270 (166 253)	30	176 769 (95 213)	−26 516	113 519	0.219

Note: USD 1 = SEK 6.50, EUR 1 = SEK 9.03 (year 2011) average exchange rate www.riksbank.se

*p < 0.05

^a^ Parents and relatives together

**Table 9:** Extended analysis of loss of production including parents’ number of days absent from work and the unit cost per day, during the period 0–24 months from diagnosis

Students’ T-test	n^a^	Mean	SD	Significance
Parents’ number of days absent from work				
*Comparisons between groups*				
Both genders				
Control group	169	8.01	11.92	
Intervention group	173	8.01	11.33	0.996
Mothers				
Control group	90	10.71	13.84	
Intervention group	90	8.26	11.42	0.196
Fathers				
Control group	79	4.92	8.33	
Intervention group	83	7.75	11.29	0.071
*Comparisons between genders*				
Both groups				
Mothers	180	9.48	12.71	
Fathers	162	6.37	10.03	0.012
Control group				
Mothers	90	10.71	13.84	
Fathers	79	4.92	8.33	0.001
Intervention group				
Mothers	90	8.26	11.42	
Fathers	83	7.75	11.29	0.796
Unit costs per day (SEK)				
*Comparison between groups*				
Both genders				
Control group	169	2855	1615	
Intervention group	173	2542	2271	0.143
*Comparisons between genders*				
Both groups				
Mothers	180	2150	936	
Fathers	162	3304	2570	0.000
Control group				
Mothers	90	2409	1119	
Fathers	79	3363	1924	0.000
Intervention group				
Mothers	90	1891	612	
Fathers	83	3248	3072	0.000

Note: USD 1 = SEK 6.50, EUR 1 = SEK 9.03 (year 2011) average exchange rate www.riksbank.se

^a^The parents included in the analysis represent the parents of totally 60 children, who were asked for information of absent from work and income for each of the four follow-up periods

### Cost effectiveness and cost utility

The cost-effectiveness and cost-utility analyses relate costs to effectiveness in terms of HbA1C and to utility in terms of SF-6D.

Since no statistically significant differences between the intervention group and the control group regarding HbA1c or SF-6D were found, and since statistically significantly lower healthcare costs were found, the results indicate that HBHC might be a cost-effective strategy in a healthcare sector perspective. However, when adding the indirect costs of lost production to the direct healthcare costs, no statistically significant difference was found. Thus, when using the wider societal perspective, no difference in cost effectiveness or cost utility was found.

## Discussion

This study compared two different regimens for the initial management of children diagnosed with type 1 diabetes; hospital-based care and hospital-based home are (HBHC). The results two years from diagnosis could not detect any statistically significant difference between the two groups as concerns efficacy and child safety in terms of metabolic control and episodes of severe hypoglycaemia. This is in line with the earlier follow-up from the same study [28, 46, 47] and with another recent retrospective study comparing different sites for initial diabetes education [17].

Even though no statistically significant difference could be detected in the present study, there might still be real but not detected differences in the population at large. When interpreting results, one must hence also take into consideration the difference that the study was powered to detect [48]. Even though our power calculations were performed prior to the study and at the time, with an estimated clinically relevant difference of HbA1c, the defined effect in HbA1c might not be a likely effect size as to expect. However, there is a balance to be made between sample size and external validity over variation in settings by the period of recruitment. Treatment manuals were used and checked regularly but healthcare evolves continuously. This was evident in the routines of a gradually increased number of outpatient visits to the diabetes nurse in the hospital-based care [28, 47]. Healthcare is inherently complex and a potential problem occurs, when results of studies that have been performed under circumstances that are not representative of the forms of care normally in practice, are generalized to uncontrolled circumstances [49, 50]. Since the intervention was intended to be implemented within clinical practice, if found to be a safe and effective way of caring for a child, there needed to be a balance between the validity of inference and the implementation of care [48].

The response rate at 24 months was lower than at previous follow-ups, probably explained by changed routines of data-collection. In the first years, a research assistant who was not involved in the care assessed the outcomes and booked appointments with families outside the hospital in order to let them answer the questionnaires. Since the inclusion of families took longer than planned, we needed to change the routines, implying that the questionnaires were sent home by mail with a return envelope to the families instead. This might have brought in variability in the final outcomes. However, the results from the 24 month follow-up are in line with the earlier follow-up of the same study. Even though no statistically significant difference could be detected in parents’ reporting on the impact of a chronic paediatric health condition and parents’ health-related quality of life, the direction of effect might indicate that the initial management and the initial support given to families have an impact on families’ future experiences of living with a child diagnosed with diabetes. Although fathers had significantly higher incomes than mothers and, hence, higher lost value of production per day absent from work in both the HBHC group and the hospital group, there was a significant difference between the two genders in the number of days absent from work only for the hospital group (in which mothers had twice the number of days of absence than fathers). In a qualitative evaluation of this RCT study [51], the and learning conditions in HBHC seemed to support partnership and collaboration. The parents in HBHC described how they became active participants in negotiations of daily management and decision making in contrast to their passive role in the hospital-based care. Although the difference among parents in the two groups may be due to some underlying factor not controlled for, an interpretation might also be that the main active component in the HBHC has supported the fostering of parents’ responsibility.

Parents in HBHC have, in the earlier follow-up consistently reported significantly greater satisfaction with healthcare compared to parents in the hospital-based care [28, 46, 47]. Despite that difference in the management between groups only involved the first month from diagnosis, parents continued to report a significantly greater level of satisfaction with the healthcare two years from diagnosis. In addition to the home-like environment, the individualised learning process was an important part of the intervention bringing a focus on normalization, supporting the family to find strategies as to achieve good glycaemic control in combination with a re-establishment of their normal lifestyle. In the qualitative evaluation of the study the hospital-based care was considered as being safe but not family- or diabetes-oriented [51]. The HBHC was described as a relaxed environment, providing individualized accessibility and possibilities for situational learning and was considered as more flexible, promoting normality and involvement. This is in line with what others have found when children and young people described inflexibility in clinic processes and the dissonance this represented as compared to families’ expectations of a normal life [6]. Parents who experienced hospital-based care when their child was diagnosed with diabetes felt as if their role as parents, the person the child could rely on, was taken away from them [14]. This made them and their families feel insecure; so holding on to the hospital routines that they initially learned [12] might be one way of feeling more in control in a distressing and life-changing event.

As for our economic analyses, it should be observed that the project included no collection of data on other direct and indirect costs besides healthcare and lost market production value, even though there might have been substantial changes in families’ use of time and other resources; see discussion above. Another drawback is the fact that sample size was not chosen with the aim to detect significant differences in costs and utility. Thus, there might be real but not detected differences in cost effectiveness and cost utility due to study design. However, since the difference in SF-6D between the two groups had as low *p*-value as 0.067, and since the healthcare satisfaction index showed a statistically significant higher value, it should be fairly safe to conclude that HBHC is a cost-effective strategy, at least in a narrow healthcare sector perspective.

In line with available estimates of the cost of diabetes disease to society at large [7, 8], we found that the indirect costs of lost production were substantially higher than the direct healthcare costs in both care groups. This fact is worth noticing, even though no significant difference could be observed between the two groups. The dominance of indirect costs also meant that no significant difference could be detected in total costs (direct and indirect costs together). So, whether HBHC might be cost-effective also in a wider societal perspective remains to be shown. Both perspectives are important for policy-makers. In some countries, the United Kingdom, for instance, government authorities recommend the healthcare perspective to be used, when allocating resources in the National Health Service [27], whereas in other counties, for instance, Swedish central government authorities use the societal perspective [26].

## Conclusions

Few studies have provided high-quality evidence when comparing hospital-based care with different models of home-based care. The results of this study support the safety and feasibility of HBHC, when a child is diagnosed with Type 1 diabetes. Our results further suggest that the initial period of care and the strategies for diabetes management that are first presented to the family may exert an impact on families’ daily living for a longer period of time. The results also suggest that HBHC is a cost-effective strategy, at least in a narrow healthcare sector perspective.

## Abbreviations

CONSORT, consolidated standards of reporting trials; HbA1c, glycated haemoglobin; HBHC, hospital-based home care; PedsQL, pediatric quality of life; RCT, randomised controlled trial; SEK, Swedish crown; SF-36, short form – 36 health survey; SF-6D, short form – 6 dimensions.
